# Bacteriuria profile and antimicrobial sensitivity among pregnant women attending antenatal care at Jazan and Sabyia general hospitals, Jazan Region, KSA: A cross‐sectional study

**DOI:** 10.1002/ijgo.70082

**Published:** 2025-03-24

**Authors:** Isameldin Elamin Medani, Ahlam Hakami, Ahmed Altraifi, Maha Murtada Abdelmageed, Ali Khormi, Uma Chourasia, Essam Falagy, Ahmad Alnamy, Bishi Moukli, Sarah Mnaa, Maha Alanazi, Manal Jamali, Ali Maashi, Hayat Khudhayr, Sara Eltigani, Nouf Shahhar, Safia Muqri, Nada Makein, Yasser Majrabi

**Affiliations:** ^1^ Department of Obstetrics and Gynecology, Faculty of Medicine Jazan University Jazan Saudi Arabia; ^2^ Department of Biochemistry Lab, Faculty of Medicine Jazan University Jazan Saudi Arabia; ^3^ Department of Laboratory and Blood Bank Sabyia General Hospital, Jazan Health Cluster Jazan Saudi Arabia; ^4^ Department of Laboratories King Fahad Hospital Hail Saudi Arabia; ^5^ Department of Obstetrics and Gynecology Jazan General Hospital, Jazan Health Cluster Jazan Saudi Arabia; ^6^ Department of Laboratory, Jazan General Hospital, Jazan Health Cluster Jazan Saudi Arabia; ^7^ Department of Family and Community Medicine Faculty of Medicine, Jazan University Jazan Saudi Arabia

**Keywords:** antibiotic resistance, asymptomatic bacteriuria, microbial sensitivity, UTI

## Abstract

**Background:**

Bacteria in urine, which is known as bacteriuria, is divided clinically into two types: symptomatic, where the patient experiences urinary complaints, and asymptomatic (ASB), in which the patient has no complaints. Pregnant women who have ASB may later develop symptomatic urinary tract infection, which is considered one of the most common bacterial infections in pregnancy and which, if untreated, can result in serious adverse pregnancy outcomes.

**Objective:**

The goal of this research was to find out the prevalence of bacteriuria and its related factors among pregnant women in the Jazan Region, Saudi Arabia, recognize correlated microbial organisms, and identify the antimicrobial profile.

**Methods:**

A cross‐sectional study was conducted at Jazan and Sabyia general hospitals from June 28, 2023 to June 28, 2024. A systematic random sample of 392 pregnant women was selected. Urine samples were collected from them for bacteriological cultures and antimicrobial sensitivity testing. Data were collected and analyzed.

**Results:**

Out of 392 participants, 19% had bacteriuria. Most women were aged 26–35, overweight, and resided in urban areas. The most common bacteria found were *Escherichia coli* (39.2%) and *Klebsiella pneumoniae* (17.6%). Antimicrobial sensitivity was highest for cefuroxime and vancomycin and lowest for erythromycin and penicillin. Significant predictors of bacteriuria were residence, body mass index, gestational age, hemoglobin levels, and hemoglobin A1c levels.

**Conclusion:**

The study revealed a significant prevalence of bacteriuria among pregnant women in Jazan Region, highlighting the importance of routine screening and targeted interventions for high‐risk groups. Effective antibiotic stewardship programs are essential to managing resistance patterns.

## 1 INTRODUCTION

Bacteriuria, the presence of bacteria in urine, is classified into symptomatic and asymptomatic types. Asymptomatic bacteriuria (ASB) involves bacterial colonization without symptoms, whereas symptomatic bacteriuria indicates a urinary tract infection (UTI). Women are more susceptible due to anatomical factors. UTIs in females often result from fecal flora entering the urinary tract.^1^ UTIs are the most common bacterial infections during pregnancy.^2^, ^3^ Furthermore, ASB occurs in (2%–10%) of all pregnancies.^4^ A study conducted in Iraq's Kurdistan found that “among the 5,042 pregnant women included in the study, significant bacteriuria was found in 625 (12.40%) of the cases, and 198 (31.68%) had symptomatic UTI, of which (43.59%) were diagnosed during the third trimester”.^5^ Around 20%–40% of pregnant women with ASB who are mistreated will eventually develop pyelonephritis later in their pregnancy, while less than 1% of those without ASB will develop it.^2^ It was found that pyelonephritis can cause adverse prenatal and maternal pregnancy outcomes such as low‐birth‐weight babies, early delivery, fetal death, pre‐eclampsia, pregnancy‐induced hypertension, anemia, thrombocytopenia, and temporary renal insufficiency.^6^, ^7^ de Souza et al. reported that “Bacterial colonization of the urinary tract during pregnancy may also be associated with adverse perinatal outcomes such as prematurity, low birth weight, premature rupture of ovular membranes, and hypertensive syndromes”.^8^ It is estimated that every other woman will have had at least one UTI during her lifetime, with (10%–60%) of all women having a symptomatic UTI at least once in their lives. The infection risk increases with age.^9^ In pregnancy, the most common pathogenic organism associated with both asymptomatic and symptomatic bacteriuria is *Escherichia coli*, which accounts for 60%–80% of all UTIs.^10^ A recommendation to screen pregnant women for bacteriuria with urine culture at 12–16 weeks of gestation was made by the US Preventive Services Task Force, targeted to identify 80% of women who will eventually develop ASB.^11^ Effective strategies for rapid and frequent surveillance are urgently needed to monitor antimicrobial resistance prevalent in low‐ and middle‐income countries.^12^ In Saudi Arabia, there are insufficient old data and no recent data about the prevalence of bacteriuria during pregnancy.^13^ There is no evidence to recommend that women should not get screened for ASB, and there are no current prevalence statistics, so that it is difficult to determine the cost‐effectiveness of doing so. The aim of this research was to determine the occurrence of bacteriuria and its related factors among pregnant women in Jazan Region. Furthermore, we determined the most common causative organisms of bacteriuria and assessed the antimicrobial susceptibility profile because of the worldwide increase in resistance to antibiotics due to their indiscriminate use.^11^

## 2 MATERIALS AND METHODS

### 2.1 Study design and setting

A cross‐sectional study was conducted at Jazan and Sabyia general hospitals in the Jazan Region in southern Saudi Arabia from June 28, 2023 to June 28, 2024.

### 2.2 Sampling method

Systematic random selection of pregnant women who attended the antenatal clinic and agreed to participate in the study was done. Informed consent was obtained from them, and a face‐to‐face interview was done. Very ill patients and those who refused to participate in the study, were excluded. All demographic data and related maternal information were obtained and entered into a structured questionnaire.

The sample size was calculated using a single population proportion formula at 15% prevalence in eastern Saudi Arabia^14^ and a 95% confidence level. The sample size was doubled for greater accuracy, amounting to a total of 392 women. Sample size was not recalculated after exclusion.

### 2.3 Laboratory procedures

A volume of 5 mL or more of midstream urine was collected from all pregnant women who participated in the study, placed in sterile containers and sent for urine microscopy, culture, and sensitivity testing. Any bacterial growth with colony counts of ≥105 CFU/mL of urine was classified as significant bacteriuria. Colony characteristics, Gram staining, and a series of biochemical tests were used to identify bacterial organisms according to the standard bacteriological procedure. Antibiotic sensitivity testing was performed using the MicroScan dilution method for fully automated identification and sensitivity according to the Clinical and Laboratory Standards Institute guidelines.^15^

### 2.4 Data analysis

The data collected from participants were coded and entered into SPSS system files (SPSS IBM 28). The data were analyzed and interpreted. The Shapiro–Wilk test and the Kolmogorov–Smirnov test were used to test the normality distribution of data. Using univariate analyses like the Mann–Whitney *U*‐test and Student *t*‐test, the significance of the quantitative variable results was assessed, while the qualitative variable significance was calculated using the Monte Carlo test, chi‐square test, and Fisher exact test.

Potential confounders were identified and controlled during data analysis using multivariable analysis to adjust for their effects.

### 2.5 Statistical analysis

Collected data were first entered into an Excel sheet and later exported to IBM SPSS 28 for the purpose of analysis. Categorical data were described using frequency tables with proportions and graphically using donut charts. Measures of central tendency were used to summarize numerical data, and the Shapiro–Wilk and Kolmogorov–Smirnov tests were used to test for normality. The numerical data in this study were not normally distributed. The prevalence of bacteriuria was calculated as a proportion. This was done by dividing the number of positive bacterial growths by the total size of the samples. To determine predictors of bacteriuria, odds ratios were calculated using the maximum likelihood estimation technique. Chi‐square or Fisher exact tests were used, as appropriate, to assess differences in the proportions of bacteriuria‐positive and bacteriuria‐negative patients. Any *P* < 0.05 between variables was considered to be statistically significant (at 95% confidence interval [CI]), and exposure variables showing such significance value are known as predictors of bacteriuria in pregnancy.

### 2.6 Steps in regression analysis

Because of the binary character of the dependent variable, a binary logistic regression model was employed for the statistical analysis to assess the factors that influence the dependent variable (bacteriuria). Exclusion of variables (e.g., occupation, nationality) was based on statistical insignificance and poor contribution to the model. All the variables unrelated to the analysis were excluded as a first step in the model‐building method. A necessary step in the model‐building strategy is the selection of the variables that fit the model. Both direct and stepwise methods were used, and it showed that some variables contributed poorly (“insignificantly”) to the model, and these were excluded from the final model. All variables with a level of significance *P* < 0.05 in the multivariable analysis were selected for the model. The −2 log‐likelihood of the model was significantly lower (minimum unexplained variation on the dependent variable) compared with several other models as well as the intercept‐only model. Furthermore, Nagelkerke *R* ^2^ was used to explain the variation in the dependent variable when applying the model. Also, the percentage of cases in which the model was correctly classified was higher than that found in several models, including the intercept‐only model. After the fit of the multivariable model, the influence of each variable in the model was verified by the Wald statistic.

## 3 RESULTS

The aim of this study was to determine the prevalence of bacteriuria in pregnancy along with the antibiotic sensitivity pattern of the organisms involved and to monitor drug resistance. A total of 392 pregnant women participated in the study—321 (81.9%) were Saudis and 71 (18.1%) were non‐Saudis, and 196 were from Jazan General Hospital and 196 pregnant women were from Sabiya General Hospital in Jazan Region, in southern Saudi Arabia. The majority of pregnant women (51.5%) were between 26 and 35 years old (mean age 31.6 ± 6.3), 84.2% were housewives and 80.9% resided in urban areas. The majority of women (70.3%) were blood group type O, Rh‐positive (94.6%). 35.7% were overweight (mean BMI [calculated as weight in kilograms divided by the square of height in meters] 28.1 ± 5.2) (Tables 1 and 2).

**TABLE 1:** Socio‐demographical characteristics of pregnant women attending Jazan General Hospital and Sabyia General Hospital in Jazan Region, Saudi Arabia, 2024 (*n* = 392).

Demographical characteristics		Frequency	Percent
Age group (years)	17–25	72	18.4
	26–35	202	51.5
	36–45	118	30.1
	(mean ± SD)	31.6 ± 6.3	
Nationality	Saudi	321	81.9
	Non‐Saudi	71	18.1
Occupation	Housewife	330	84.2
	Employee	62	15.8
	Total	392	100.0
Blood group	O	52	70.3
	A	14	18.9
	B	7	9.5
	AB	1	1.4
Rh	Negative	4	5.4
	Positive	70	94.6

Abbreviations: Rh, Rhesus blood group.

**TABLE 2:** Socio‐demographic and pregnancy characteristics associated with bacteriuria among pregnant women attending Jazan General Hospital and Sabyia General Hospital in Jazan Region, Saudi Arabia, 2024 (*n* = 392).

Variables	Tested (*n* [%])	Bacteriuria		Total (%)	*χ* ^2^, *P*‐value
		Absent (*n* = 318)	Present (*n* = 74)		
Hospital	Jazan Hospital	175 (89.3)	21 (10.7)	196 (50)	0.000***
	Sabyia Hospital	143 (73)	53 (27)	196 (50)	
Residence	Urban	267 (84.2)	50 (15.8)	317 (80.9)	0.000***
	Rural	51 (68)	24 (32)	75 (19.1)	
BMI	Normal	5 (83.3)	1 (16.7)	6 (1.5)	0.000***
	Underweight	117 (97.5)	3 (2.5)	120 (30.6)	
	Overweight	97 (9.3)	43 (30.7)	140 (35.7)	
	Obese	99 (78.6)	27 (21.4)	126 (32.1)	
Parity	Nullipara	63 (71.6)	25 (28.4)	88 (22.4)	0.006**
	Primipara	46 (75.4)	15 (24.6)	61 (15.6)	
	Multipara	209 (86)	34 (14)	243 (62.0)	
Gestational age	First trimester	40 (62.5)	24 (37.5)	64 (16.3)	0.000***
	Second trimester	109 (77.9)	31 (22.1)	140 (35.7)	
	Third trimester	169 (89.9)	19 (10.1)	188 (48.0)	
Hb level	Normal	189 (89.6)	22 (10.4)	211 (53.8)	0.000***
	Anemic	129 (71.3)	52 (28.7)	181 (46.2)	
HbA1c	Normal	296 (87.8)	41 (12.2)	337 (86.0)	0.000***
	High	22 (40)	33 (60)	55 (14.0)	
Total (%)		318 (81.1)	74 (18.9)	392 (100)	

Abbreviations: BMI, body mass index (calculated as weight in kilograms divided by the square of height in meters); Hb, hemoglobin; HbA1c, hemoglobin A1c; *n*, frequency.

** *P* < 0.01.

*** *P* < 0.001.

The pregnancy characteristics of patients with bacteriuria showed that most of them were in their second (41.9%) and first trimesters (32.4%) (mean gestational age 20.1 ± 9.6 weeks), and 45.9% were multiparous. According to their medical history, 21.6% had a history of hypertension, 20.3% had diabetes, and 10.8% had a history of sexually transmitted disease. Moreover, 13.5% were using treatment such as amoxicillin, ampicillin, ceftriaxone, cefuroxime, and nystatin (Tables 2 and 3).

**TABLE 3:** Type of organisms associated with bacteriuria among pregnant women attending Jazan General Hospital and Sabyia General Hospital in Jazan Region, Saudi Arabia, 2024 (*n* = 74).

Type of organism	Overall bacteriuria	
	Frequency	Percent
*Acinetobacter lwoffii*	3	4.1
*Candida albicans*	4	5.4
*Candida tropicalis*	1	1.4
*Escherichia coli*	29	39.2
*Enterococcus faecalis*	2	2.7
*Enterobacter cloacae*	1	1.4
*Klebsiella pneumoniae*	13	17.6
*Koceria kristina*	1	1.4
*Mixed*	5	6.8
*Pseudomonas aeruginosa*	1	1.4
*Staphylococcus haemolyticus*	1	1.4
*Staphylococcus sciuri*	1	1.4
*Staphylococcus agalactiae*	2	2.7
*Staphylococcus aureus*	4	5.4
*Staphylococcus epidermidis*	1	1.4
*Staphylococcus lentus*	1	1.4
*Staphylococcus saprophyticus*	1	1.4
*Staphylococcus xylosus*	1	1.4
*Streptococcus agalactiae*	2	2.7
Total	74	100.0

The laboratory investigations showed that 70.3% had low hemoglobin (Hb) levels (anemic <11 mg/dL; mean Hb level 9.5 ± 1.0); 39.2% presented with high hemoglobin A1c (HbA1c) levels (mean HbA1c 6.1 ± 0.8) and 24.3% with high TWBCs (total white blood cell count) (mean TWBCs 8586.7 cells/µL ± 4021.0). Moreover, urine generally showed red blood cells (RBCs) in 37.8% of the samples, casts and protein in 6.8%, pyuria in 77%, and bacteria in urine in 100%. Microscopically, bacterial count in urine was (1+) 58%, and (2+) 24.6%, (Table 3).

**FIGURE 1:**
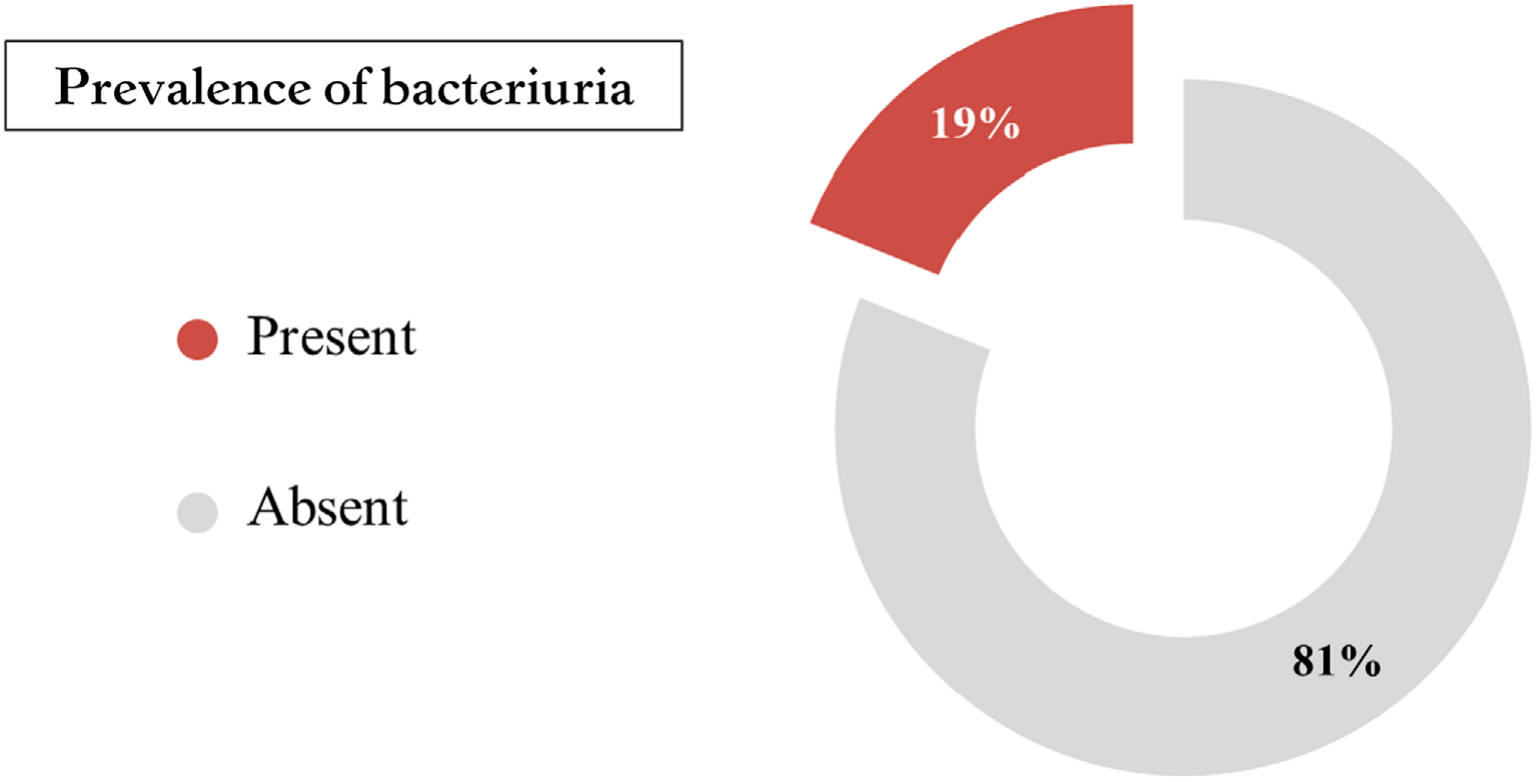
Prevalence of bacteriuria among pregnant women attending Jazan General Hospital and Sabyia General Hospital, Jazan Region, Saudi Arabia, 2024 (*n* = 392).

**FIGURE 2:**
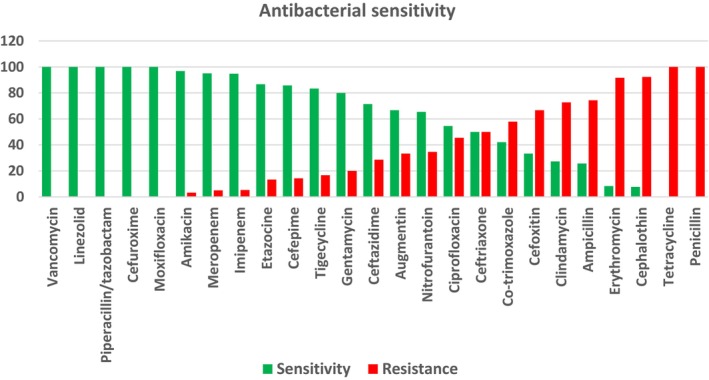
Antibacterial sensitivity among pregnant women with bacteriuria (*n* = 74).

Out of 392 pregnant women who underwent bacteriological investigations, 19% were found to have bacteriuria (Figure 1). The majority of the bacterial organisms were *E. coli* (39.2%), *K. pneumoniae* (17.6%), *Staphylococcus agalactiae*, and *Candida albicans* (5.4%) (Table 3). Regarding the antimicrobial sensitivity profile, the overall sensitivity among patients with bacteriuria (*n* = 74) was 66%, compared with resistance for 34%. The organisms isolated were most sensitive (100%–95%) to vancomycin, linezolid, piperacillin/tazobactam, cefuroxime, moxifloxacin, amikacin, and meropenem and imipenem; next were etazocine, cefepime, tigecycline, gentamicin, ceftazidime, Augmentin (amoxicillin/clavulanic), nitrofurantoin, and ciprofloxacin, with sensitivities of between 55% and 90%. Others with lower sensitivity were co‐trimoxazole, cefoxitin, clindamycin, and ampicillin (42%–26%), while the least sensitive (0%–8%) were erythromycin, cephalothin, tetracycline, and penicillin. The extremely low sensitivity to erythromycin (0%–8%) and penicillin (0%–8%) could have a significant impact on empirical therapy choices in the region. These low sensitivity rates suggest that these antibiotics may not be effective for treating bacteriuria in pregnant women in this area, emphasizing the need for updated and region‐specific antibiotic guidelines (Figure 2; Table 4).

**TABLE 4:** Antibacterial sensitivity against bacterial isolates of pregnant women with bacteriuria attending Jazan General Hospital and Sabyia General Hospital in Jazan Region, Saudi Arabia, 2024 (*n* = 74).

Description	Antibiotic	No Sensitive	Sensitivity	Resistant	Resistance	Total
Most common	Vancomycin	14	100	0	0	14
	Linezolid	9	100	0	0	9
	Piperacillin/tazobactam	7	100	0	0	7
	Cefuroxime	4	100	0	0	4
	Moxifloxacin	4	100	0	0	4
	Amikacin	30	97	1	3	31
	Meropenem	19	95	1	5	20
	Imipenem	18	95	1	5	19
	Etazocine	13	87	2	13	15
	Cefepime	12	86	2	14	14
	Tigecycline	5	83	1	17	6
	Gentamycin	20	80	5	20	25
	Ceftazidime	5	71	2	29	7
	Augmentin	30	67	15	33	45
	Nitrofurantoin	17	65	9	35	26
	Ciprofloxacin	12	55	10	45	22
	Ceftriaxone	8	50	8	50	16
	Co‐trimoxazole	8	42	11	58	19
	Cefoxitin	3	33	6	67	9
	Clindamycin	3	27	8	73	11
	Ampicillin	9	26	26	74	35
	Erythromycin	1	8	11	92	12
	Cephalothin	1	8	12	92	13
	Tetracycline	0	0	5	100	5
	Penicillin	0	0	5	100	5
Least common	Aztreonam	0	0.0	2	100	2
	Cefazoline	2	100	0	0.0	2
	Cefotaxime	1	33.3	2	66.6	3
	Ceftazidime	3	60	2	40	5
	Ertapenem	0	0.0	2	100	2
	Levofloxacin	0	0.0	3	100	3
	Oxacillin	0	0.0	1	100	1
	Rifampicin	0	0.0	1	100	1
	Tobramycin	0	0.0	2	100	2
	Trimethoprim	0	0.0	3	100	3

*Escherichia coli* (*n* = 29) was most sensitive to amikacin (72%), Augmentin (52%), imipenem, and meropenem (41%), and resistant to ampicillin (41%), cephalothin (31%), ciprofloxacin (28%), co‐trimoxazole (24%), and ceftriaxone (24%). *K. pneumoniae* (*n* = 13) was mostly sensitive to amikacin (46%), gentamicin (46%), meropenem (46%), and cefepime (38%), while being resistant to ampicillin (77%), Augmentin (54%), and nitrofurantoin (31%). *S. aureus* (*n* = 4) was highly sensitive to gentamicin (100%) and vancomycin (75%) and highly resistant to erythromycin (100%), ampicillin (50%), and clindamycin (50%) (see Appendix “Antibacterial sensitivity profile”).

Applying chi‐square and Fisher exact tests, hospital, residence, BMI, parity, gestational age, Hb, and HbA1c levels were all significantly associated with bacteriuria (*P* < 0.05) (Table 5). Moreover, binary logistic regression was performed to ascertain the effects of hospital setting, residence, BMI, gestational age, Hb, HbA1c, and number of children on the likelihood that participants have bacteriuria. Using the stepwise method, occupation and nationality were deemed insignificant and hence were excluded from the model. The logistic regression model was statistically significant (*χ* ^2^(7) = 143.07, *P* < 0.001). The model explained 49.3% (Nagelkerke *R* ^2^) of the variance in bacteriuria and correctly classified 86.5% of cases.

**TABLE 5:** Regression analysis to predict bacteriuria based on associated factors among pregnant women attending Jazan General Hospital and Sabyia General Hospital in Jazan Region, Saudi Arabia, 2024 (*n* = 392).

Bacteriuria, Ref = negative	*B* (SE)	95% CI for odds ratio		
		Lower	Odds ratio	Upper
Hospital (Ref = Jazan)	1.71 (0.40)**	2.53	5.52	12.05
Residence (Ref = urban)	−0.82 (0.39)*	1.07	2.28	4.9
BMI	0.13 (0.04)***	1.06	1.14	1.22
Gestational age	−0.06 (0.02)**	0.91	0.94	0.98
Hb level	−0.53 (0.12)***	0.46	0.59	0.75
HbA1c	1.24 (0.20)***	2.32	3.44	5.09
No. of children	−0.22 (0.09)*	0.67	0.80	0.96
Intercept	−5.49 (1.81)**			

*Note*: Nagelkerke *R* ^2^ = 0.493, model χ^2^(7) = 143.07, *P* < 0.001.

Abbreviations: *B*, coefficient; BMI, body mass index (calculated as weight in kilograms divided by the square of height in meters); CI, confidence interval; Hb, hemoglobin; HbA1c, hemoglobin A1c; Ref, baseline/reference; SE, standard error.

* *P* < 0.5.

** *P* < 0.01.

*** *P* < 0.001.

This suggests the possibility of omitted variables that could further explain the likelihood of bacteriuria. Future studies might consider additional predictors such as socioeconomic status or detailed parity history to improve the model's explanatory power.

Pregnant women who attended Sabiya General Hospital were 5.52 times more likely to exhibit bacteriuria than those who attended Jazan General Hospital. Those from rural areas were 2.28 times more likely to have bacteriuria than those in urban areas. Increasing BMI and HbA1c were associated with an increased likelihood of exhibiting bacteriuria, but increasing gestational age, Hb level, and number of children were associated with a reduction in the likelihood of acquiring bacteriuria (Table 5).

## 4 DISCUSSION

The present study aimed to determine the prevalence of bacteriuria among pregnant women and to analyze the antibiotic sensitivity patterns of the organisms involved. The prevalence of bacteriuria in pregnant women was found to be 19%, which is consistent with previous studies indicating that bacteriuria is a common condition during pregnancy. The study also identified significant associations between bacteriuria and several socio‐demographic and clinical factors, including hospital setting, residence, BMI, gestational age, Hb levels, and HbA1c levels.

The logistic regression analysis revealed that pregnant women attending Sabiya General Hospital were significantly more likely to exhibit bacteriuria compared to those attending Jazan General Hospital. This could be due to differences in patient populations, healthcare practices, or environmental factors between the two hospitals. Additionally, women residing in rural areas had a higher likelihood of bacteriuria compared with those in urban areas, possibly due to differences in access to healthcare and sanitation.

The study found that increasing BMI and HbA1c levels were associated with a higher likelihood of bacteriuria, while increasing gestational age, Hb levels, and the number of children were associated with a reduced likelihood. Future studies should investigate whether this association is primarily driven by better overall health or enhanced prenatal care in later trimesters. These findings suggest that overweight and diabetic pregnant women may be at higher risk for bacteriuria, highlighting the need for targeted screening and intervention in these populations.

In this study, bacteriuria was more likely in the second trimester in multiparous, anemic, and high HbA1c patients. There was higher prevalence in rural areas and in Sabiya Hospital. For instance, the significant prevalence of bacteriuria among pregnant women in the Jazan Region aligns with previous studies, such as those conducted by Ezugwu et al. in Nigeria and Mokube et al. in Cameroon, which also reported high prevalence rates and identified common pathogens such as *E. coli* and *K. pneumoniae*.^16^, ^17^ In Nigeria, Ezugwu et al. showed that UTI was more prevalent in older, married, third‐trimester, higher‐parity, low‐education women with untreated previous UTIs, diabetes, and hypertension.^16^ Both studies highlight the significant prevalence of bacteriuria/UTI among pregnant women, with notable differences in demographic and clinical characteristics influencing the likelihood of infection. In comparison with the Mokube et al. study, which was conducted in Buea, Cameroon, both studies had a majority of participants in their late 20s to early 30s, but the Mokube et al. study had a younger cohort and a higher education level.^17^ Both studies reported a similar prevalence of bacteriuria. This study identified significant predictors, while Mokube et al. did not find statistically significant predictors but noted trends. Both studies found *E. coli* and *K. pneumoniae* to be the most common isolates; Mokube et al.'s study had a more diverse range of bacteria and reported higher overall sensitivity rates to a broader range of antibiotics compared with this study.^17^

The antibiotic sensitivity profile indicated that Augmentin, amikacin, and gentamicin were among the most effective antibiotics against the bacterial isolates, while ampicillin, Augmentin, and cephalothin showed higher resistance rates. *E. coli*, the most common isolate, exhibited significant sensitivity to amikacin and Augmentin but also showed resistance to several antibiotics, including ampicillin and Augmentin. These findings underscore the importance of routine antibiotic sensitivity testing to guide effective treatment and combat antibiotic resistance.

For antibiotic sensitivity, this study focused on sensitivity profiles, while Al‐Kashif highlighted the most widely used antibiotics.^13^ Both studies identified different significant factors, with some overlap, such as previous UTI and clinical manifestations. They provide complementary insights into bacteriuria in pregnant women, highlighting differences in prevalence, common pathogens, antibiotic usage, and significant predictive factors. Chi‐square and Fisher exact tests revealed significant associations between bacteriuria and factors such as hospital, residence, BMI, parity, gestational age, hemoglobin, and HbA1c levels (*P* < 0.05). The logistic regression model was statistically significant (*χ* ^2^(7) = 143.07, *P* < 0.001) and explained 49.3% of the variance in bacteriuria, correctly classifying 86.5% of cases. This underscores the robustness of the identified predictors (Table 5). These findings suggest targeted interventions for high‐risk groups, including those with higher BMI and diabetes, and highlight the importance of tailored public health strategies to address disparities in healthcare access between rural and urban areas.

Overall, the study highlights the prevalence of bacteriuria in pregnancy and the importance of monitoring antibiotic resistance patterns. The findings suggest that targeted screening and tailored antibiotic therapy are crucial for managing bacteriuria in pregnant women, particularly those with higher BMI and HbA1c levels. Further research is needed to explore the underlying factors contributing to the observed differences between hospitals and to develop strategies for preventing and managing bacteriuria in pregnancy.

## 5 CONCLUSION

The study reveals a significant prevalence of bacteriuria among pregnant women in the Jazan Region, highlighting the importance of routine screening and targeted interventions for high‐risk groups. Effective antibiotic stewardship programs are essential to managing resistance patterns. It provides insights into the epidemiology of bacteriuria in pregnant women in the Jazan Region and underscores the need for targeted public health strategies and effective clinical management to address this issue.

## AUTHOR CONTRIBUTIONS

IEM: project development, manuscript editing; AH: wrote the final draft; AA: wrote the final draft of the article; MM: manuscript writing; AK: manuscript editing; UC: methodology and data analysis; EF: provided support with the logistics; AA: data collection; BM: data collection; SM: data collection; MA: data collection and management; MJ: data collection and management; AM: designed this study; HK: wrote the final draft of the article; SE: wrote the final draft; NS: data collection; SM: data collection; YM: data analysis.

## FUNDING INFORMATION

This research was funded by the deanship of scientific research at Jazan University; grant number RUP2‐06 covered the A.P.C. for publication.

## CONFLICT OF INTEREST STATEMENT

The authors declare no conflicts of interest.

## Supporting information


Appendix S1.

Appendix S2.

## Data Availability

Research data are not shared.
