# Supplementary figures and images for: Bacteriuria profile and antimicrobial sensitivity among pregnant women attending antenatal care at Jazan and Sabyia general hospitals, Jazan Region, KSA: A cross‐sectional study
Source: Int J Gynaecol Obstet. 2025 Mar 24;170(2):751–9. doi: 10.1002/ijgo.70082 (PMC12255912; doi:10.1002/ijgo.70082)

**Appendix …: Antibacterial Sensitivity Profile 1**


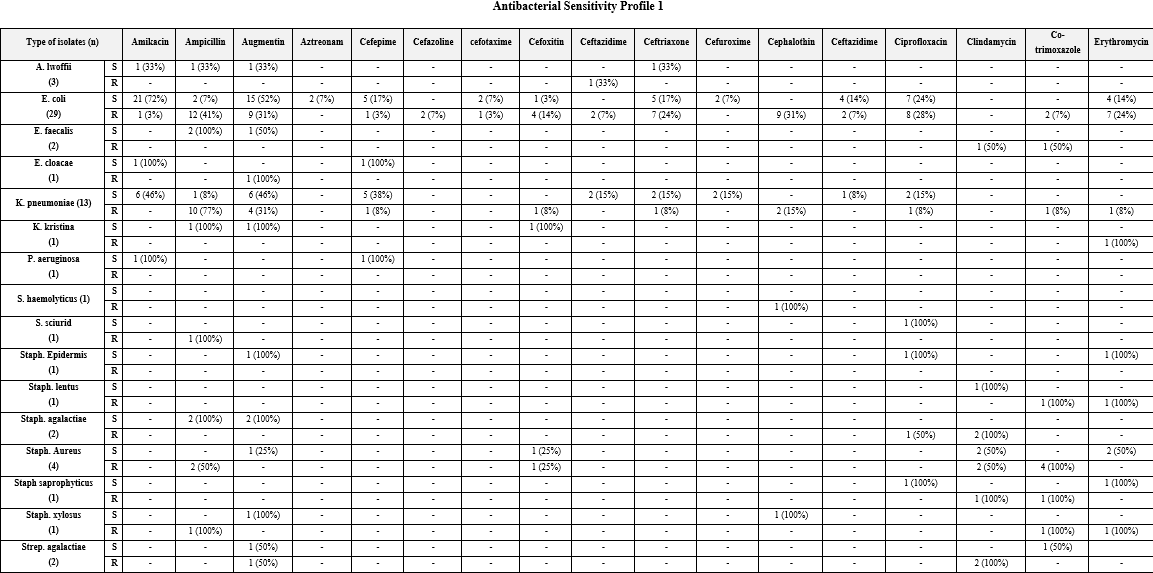


**Antibacterial Sensitivity Profile 2**


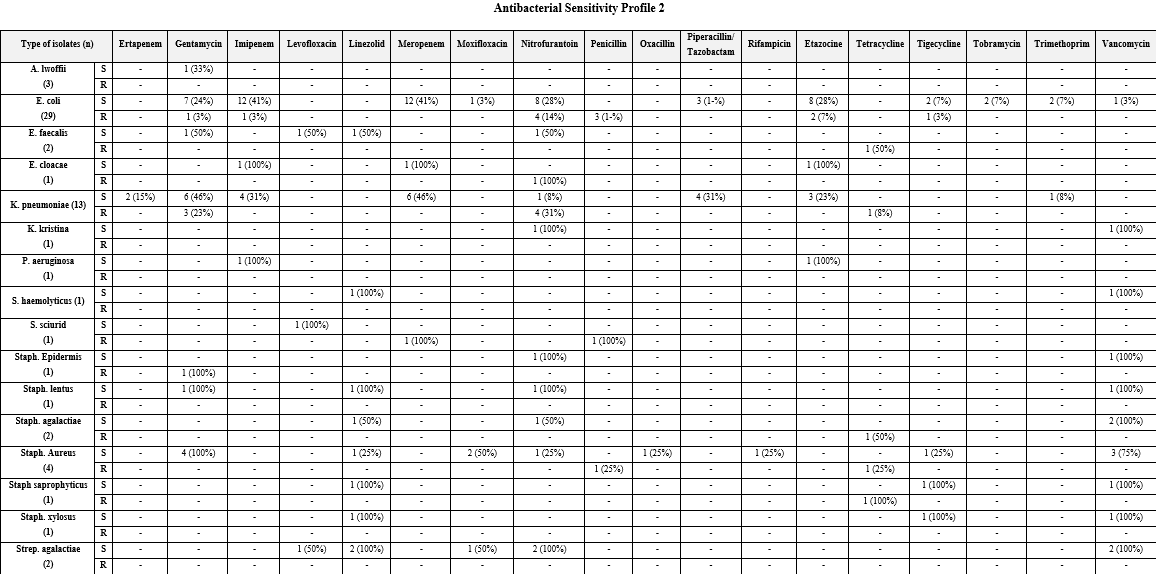

Supplement: Supplementary file 1 — Appendix S1. [file IJGO-170-751-s002.docx]
